# Genetic labeling reveals temporal and spatial expression pattern of D2 dopamine receptor in rat forebrain

**DOI:** 10.1007/s00429-018-01824-2

**Published:** 2019-01-02

**Authors:** Qing Yu, Ying-Zi Liu, Yan-Bing Zhu, Yao-Yi Wang, Qian Li, Dong-Min Yin

**Affiliations:** 1 Key Laboratory of Brain Functional Genomics, Ministry of Education, Shanghai Key Laboratory of Brain Functional Genomics, School of Life Science, East China Normal University, Shanghai, 200062 China; 2 Experimental and Translational Research Center, Beijing Friendship Hospital, Capital Medical University, Beijing, China; 3 Neuroscience Division, Department of Anatomy and Physiology, Shanghai Jiao Tong University School of Medicine, Shanghai, China

**Keywords:** Drd2, Knockin rats, Olfactory bulb, Cerebral cortex, Hippocampus

## Abstract

The D2 dopamine receptor (Drd2) is implicated in several brain disorders such as schizophrenia, Parkinson’s disease, and drug addiction. Drd2 is also the primary target of both antipsychotics and Parkinson’s disease medications. Although the expression pattern of Drd2 is relatively well known in mouse brain, the temporal and spatial distribution of Drd2 is lesser clear in rat brain due to the lack of Drd2 reporter rat lines. Here, we used CRISPR/Cas9 techniques to generate two knockin rat lines: Drd2::Cre and Rosa26::loxp-stop-loxp-tdTomato. By crossing these two lines, we produced Drd2 reporter rats expressing the fluorescence protein tdTomato under the control of the endogenous Drd2 promoter. Using fluorescence imaging and unbiased stereology, we revealed the cellular expression pattern of Drd2 in adult and postnatal rat forebrain. Strikingly, the Drd2 expression pattern differs between Drd2 reporter rats and Drd2 reporter mice generated by BAC transgene in prefrontal cortex and hippocampus. These results provide fundamental information needed for the study of Drd2 function in rat forebrain. The Drd2::Cre rats generated here may represent a useful tool to study the function of neuronal populations expressing Drd2.

## Introduction

Dopamine signaling plays critical roles in neural development and adult brain function such as locomotion, reward, learning and memory (Money and Stanwood 2013; Schultz 2007). The brain contains two types of dopamine receptors based on sequence homology and function: the excitatory D1-like receptors (D1 and D5) and inhibitory D2-like receptors (D2, D3, and D4). While all the dopamine receptors are important for brain homeostasis, D2 dopamine receptor (Drd2) is closely related with brain disorders including schizophrenia (Wong et al. 1986), Parkinson’s disease (Chaudhuri and Schapira 2009) and drug addiction (Volkow et al. 2007). Moreover, Drd2 is the primary target of antipsychotics and Parkinson’s disease medications (Beaulieu and Gainetdinov 2011; Roth et al. 2004). Determining the temporal and spatial expression pattern of Drd2 is a prerequisite for understanding its physiological function and pathophysiological roles in brain diseases.

The traditional studies on the expression pattern of Drd2 in rodent brain include those from autoradiography, in situ hybridization and immunohistochemistry. The autoradiographic experiments used H^3^-labeled DRD2 agonists or antagonists to reveal the DRD2-binding sites in the brain (Boyson et al. 1986; Charuchinda et al. 1987; Mansour et al. 1990; Yokoyama et al. 1994). The results of ligand-binding autoradiography, sometimes, are challenged by the current knowledge of receptor selectivity. For example, the study from Charuchinda et al. used a ligand found to be bind additionally to D3 receptor (Landwehrmeyer et al. 1993). The studies from in situ hybridization (ISH) used Drd2 probes to investigate the expression pattern of Drd2 mRNA (Gaspar et al. 1995; Le Moine and Gaspar 1998; Mengod et al. 1989; Weiner et al. 1991). Immunohistochemistry (IHC) analysis took use of commercial or home-made DRD2 antibodies to examine the expression pattern of DRD2 proteins (Khan et al. 1998; Lavian et al. 2018; Levey et al. 1993; Sesack et al. 1994; Stojanovic et al. 2017).

The recently developed techniques of genetic labeling have enabled clear visualization of Drd2 at the cellular level. These techniques include the use of transgenic mice expressing either the fluorescent protein GFP (Drd2-GFP) or the Cre recombinase (Drd2-Cre), under the control of Drd2 promoter (Gangarossa et al. 2012; Gong et al. 2003; Heiman et al. 2008; Puighermanal et al. 2015; Wei et al. 2018). Most genetic labeling studies have come from mouse Cre lines (Madisen et al. 2010) because of the availability of the vast genetic toolbox for mice. However, rats have unique advantages over mice. With larger brains and ability to perform more complex behavioral paradigms, rats can provide a better translational validity for brain disorders (Ellenbroek and Youn 2016). Unfortunately, the investigation of Drd2 expression in rat brain has relied on the traditional methods. However, the newly developed technique CRISPR/Cas9 has made it convenient to do gene editing in rats (Li et al. 2013).

In this study, we used the CRISPR/Cas9 technique to generate Drd2 reporter rats which express tdTomato under the control of endogenous Drd2 promoter. The expression of Drd2 is well studied in striatum medium-spiny neurons and midbrain dopaminergic neurons (Gerfen et al. 1990; Sesack et al. 1994). Here, we used Drd2 reporter rats to study the temporal and spatial expression pattern of Drd2 in forebrain regions including the olfactory bulb, cerebral cortex, and hippocampus. Our data provide a fundamental framework for the study of Drd2 function in rat forebrain. In addition, the Drd2::Cre rats generated here may represent a useful tool to study the function of neuronal populations expressing Drd2.

## Materials and methods

### Generation of Drd2::Cre knockin rats

A P2A-Cre cassette was placed between the coding sequence of exon 7 and 3′UTR of the Drd2 gene using CRISPR/Cas9 technology. The detail of using CRISPR/Cas9 technology to do gene editing in rats was described previously (Li et al. 2013). Briefly, gRNA, Cas9 mRNA, and targeting vectors were injected into the cytoplasm of one-cell stage embryos through the injection needle. Injections were performed using an Eppendorf transferMan NK2 micromanipulator. Injected zygotes were transferred into pseudopregnant female SD rats after 2-h culture in KSOM medium.

This strain was generated in Beijing Biocytogen Co., Ltd., and maintained on a Sprague Dawley genetic background. The F0 chimera rats were crossed with WT rats to get the germline transmission F1 rats. The correct targeting of the P2A-Cre in F1 rats was confirmed by southern blot and gene sequencing. The primers for genotyping the wt Drd2 allele are as follows: forward: 5′ acctagtccagtctttccttcgcct 3′; reverse: 5′ aagataccagtctccctggccctac 3′. The primers for genotyping the Drd2::Cre allele are as follows: forward: 5′ acctagtccagtctttccttcgcct 3′; reverse: 5′ cgatccctgaacatgtccatcag 3′. The PCR products for wt Drd2 and Drd2::Cre alleles were 456 and 428 bp, respectively.

### Generation of Rosa26::LSL-tdTomato knockin rats

A designed targeting sequence containing pCAG-loxP-3*STOP-loxP-tdTomato-WPRE-bGHpA was inserted in Rosa26 between exon1 and exon2 using the CRISPR/Cas9 technology. This strain was generated in Beijing Biocytogen Co., Ltd., and maintained on a Sprague Dawley genetic background. The F0 chimera rats were crossed with WT rats to get the germline transmission F1 rats. The correct targeting of the pCAG-loxP-3*STOP-loxP-tdTomato-WPRE-bGHpA in F1 rats was confirmed by southern blot and gene sequencing. The primers for genotyping the wt Rosa 26 allele are as follows: forward: 5′ ttgtattggagacaagaagcacttgctc 3′; reverse: 5′ ttgatagggctgcagaagtgggag 3′. The primers for genotyping the tdTomato KI Rosa 26 allele are as follows: forward: 5′ ttgtattggagacaagaagcacttgctc 3′; reverse: 5′ agtccctattggcgttactatgg 3′. The PCR products for wt and tdTomato KI Rosa 26 alleles were 646 and 429 bp, respectively.

### Generation of Drd2 reporter rats

The founding F1 rats were backcrossed with WT rats for five generations. The F6 heterozygous Drd2::Cre and Rosa26::LSL-tdTomato rats were crossed to get the Drd2::Cre^+/−^; Rosa26::LSL-tdTomato^+/−^ rats (abbreviated to Drd2 reporter rats). The tdTomato was specifically expressed in the cells with Cre activity which was controlled by the promoter of Drd2 gene. Cre dependent expression of tdTomato has recently been used to study the expression pattern of genes with a high temporal and spatial resolution (Bean et al. 2014; Madisen et al. 2010). Rats were housed at 23 °C with a 12 h light/dark cycle and food and water available ad libitum. Both female and male Drd2 reporter rats were used and showed similar Drd2 expression pattern in forebrain regions. For the quantification, at least three different rats or mice were used for each group.

### Generation of Drd1::Cre knockin rats

The rat Drd1 gene only has one exon. A P2A-Cre cassette was placed between the coding sequence of exon 1 and 3′UTR of the Drd1 gene using CRISPR/Cas9 technology. The Drd1::Cre knockin rat strain was generated in Beijing Biocytogen Co., Ltd., and maintained on a Sprague Dawley genetic background. The F0 chimera rats were crossed with WT rats to get the germline transmission F1 rats. The correct targeting of the P2A-Cre in F1 rats was confirmed by southern blot and gene sequencing. The primers for genotyping the wt Drd1 allele are as follows: forward: 5′ caacaatggggctgtggtgttttcc 3′; reverse: 5′ tactcccaaactgatttcagagccg 3′. The primers for genotyping the Drd1::Cre allele are as follows: forward: 5′ caacaatggggctgtggtgttttcc 3′; reverse: 5′ cgatccctgaacatgtccatcag 3′. The PCR products for wt Drd1 and Drd1::Cre alleles were 424 and 429 bp, respectively.

The founding F1 Drd1::Cre rats were backcrossed with WT rats for five generations. The F6 heterozygous Drd1::Cre and Rosa26::LSL-tdTomato rats were crossed to get the Drd1::Cre^+/−^; Rosa26::LSL-tdTomato^+/−^ rats (abbreviated to Drd1 reporter rats). The Drd1 reporter rats were used as controls in the present studies.

### Drd2 reporter mice

The Drd2 reporter mice were obtained by crossing the Drd2-Cre mice generated by BAC transgene (Gong et al. 2003) and the Rosa26::LSL-tdTomato (Ai 14) mice (Madisen et al. 2010). Both the Drd2-Cre and Ai 14 mice were heterozygous. The 2-month-old Drd2 reporter mice were used to compare with Drd2 reporter rats with regard to the Drd2 expression pattern in the forebrain. Mice were housed at 23 °C with a 12 h light/dark cycle and food and water available ad libitum. The Drd2 reporter mice were crossed with GAD67::GFP mice (Tamamaki et al. 2003) to visualize Drd2 expression in GABAergic interneurons.

### Analysis of Drd2-positive cells in brain slices

After being anesthetized with euthatal (From Merck) (60 mg/kg), rats were transcardially perfused with PBS (2 ml/g of body weight), followed by 4% PFA in PBS. Brains were harvested, incubated in 4% PFA overnight, and dehydrated at 4 °C in two steps with 20% and 30% sucrose in PBS. Brains were frozen in OCT (catalog #14-373-65; Fisher) and sectioned into 40 µm (60 µm for brain slices from P2 pups) slices on a cryostat microtome (Bosch Microm HM550) at − 20 °C. Images were taken on a Leica TCS SP8 scanning confocal microscopy.

Unbiased stereology (Tissue Gnostics, Vienna, Austria) (Guglielmetti et al. 2016; Kempf et al. 2014; Wang et al. 2015) was applied to Drd2^+^ cell counting in brain slices. To get a better understanding of the Drd2 expression profile during development, we compared the percentage of Drd2^+^ neurons in total neurons from different aged rat forebrain regions. By contrast, for comparison the Drd2 expression among different brain regions of adult rats, we count the density of Drd2^+^ cells. The density was calculated from at least ten continuous sections in Z-stack. The glia-like cells in the somatosensory cortex were manually excluded when counting the Drd2^+^ neuron number.

For adult Drd2 reporter rats, coronal brain slices from 5.9 to 6.7 mm relative to bregma were used to access olfactory bulb (OB), 4.7–4.2 mm relative to bregma were used to access medial prefrontal cortex (mPFC); − 2.8 to − 3.3 mm relative to bregma were used to access somatosensory cortex (SSC) and dorsal hippocampus; − 5.6 to − 6.04 mm relative to bregma were used to access entorhinal cortex (EC) and ventral hippocampus.

### Fluorescence in situ hybridization (FISH)

FISH for Drd2 mRNA expression was performed manually using the RNAscope Multiplex Fluorescent Reagent Kit v2 (Advanced Cell Diagnostics, Inc., Hayward, CA, USA) following the manufacturer’s instruction. The RNAscope probes targeting Drd2 and tdTomato were developed by Advanced Cell Diagnostics. The reference number of the Drd2 and tdTomato probes was 315,641 and 317,041, respectively.

### Immunofluorescence labeling

The process of immunofluorescence analysis was performed as described by our previous studies (Yin et al. 2013a). Briefly, brain slices were permeabilized with 0.3% Triton-X 100 and 5% BSA in PBS and incubated with primary antibodies at 4 °C overnight. The brain slices were not treated with Triton-x 100 when staining with anti-GAD67 antibodies. After washing with PBS for three times, samples were incubated with Alexa Fluor-488 or -405 secondary antibodies (1:1000, Invitrogen) for 1 h at room temperature. Samples were mounted with Vectashield mounting medium (Vector) and images were taken by Leica TCS SP8 confocal microscope. The following primary antibodies were used: rabbit anti-Drd2 (1:200, Millipore, AB5084P), rabbit anti-NeuN (1:500, Abcam, ab177487), mouse anti-PV (1:500, Sigma, P3088), mouse anti-GAD67 (1:300, Millipore, MAB5406), mouse anti-TBX21 (1:100, Abcam, ab91109), and rabbit anti-OMP (1:200, Abcam, ab183947).

### Western blot

The western blot was performed as described previously (Yin et al. 2013a). Homogenates of striatum were prepared in RIPA Buffer containing 50 mM Tris–HCl, pH 7.4, 150 mM NaCl, 2 mM EDTA, 1% sodium deoxycholate, 1% SDS, 1 mM PMSF, 50 mM sodium fluoride, 1 mM sodium vanadate, 1 mM DTT, and protease inhibitors cocktails. Homogenates were resolved on SDS/PAGE and transferred to nitrocellulose membranes, which were incubated in the TBS buffer containing 0.1% Tween-20 and 5% milk for 1 h at room temperature before the addition of primary antibody for incubation overnight at 4 °C. After wash, the membranes were incubated with HRP-conjugated secondary antibody in the same TBS buffer for 1 h at room temperature. Immunoreactive bands were visualized by ChemiDocTM XRS + Imaging System (BIO-RAD) using enhanced chemiluminescence (Pierce) and analyzed with Image J (NIH). The following antibodies were used: rabbit anti-DRD2 (1:200, Millipore, AB5084P) and mouse anti-GAPDH (1:8000, Arigo, ARG10112).

### Stereotaxic adeno-associated virus (AAV) injection

The pAAV-EF1a-loxp-stop-loxp-tdTomato-WPRE-poly A was generated by Obio Technology (Shanghai) Corp., Ltd. The titer of AAV is 10^13^/µl and we injected 0.5 µl AAV into each brain region. Adult rats (2-month-old) were anesthetized with euthatal (60 mg/kg, i.p. injection) and head-fixed in a stereotaxic device (RWD life science). Injection coordinates are as follows: anteroposterior (AP) 6.70 mm, dorsoventral (DV) 3.20 mm, mediolateral (ML) 1.50 mm relative to bregma for olfactory bulb; AP 1.00 mm, DV 5.00 mm, and ML 2.50 mm relative to bregma for striatum; AP − 6.30 mm, DV 8.20 mm, and ML 5.00 mm relative to bregma for entorhinal cortex.

### Statistics

All the data were shown as mean ± SEM. Comparisons between two groups were made using unpaired *t* test. Comparisons between three or more groups were made using one-way ANOVA analysis followed by Tukey’s post hoc test. Comparison between different layers of cortex from different aged rats was performed by two-way ANOVA. Data marked with asterisks were significantly different from the control as follows: ***p < 0.001, **p < 0.01, and *p < 0.05.

## Results

### Generation and validation of Drd2 reporter rats

**Fig. 1:**
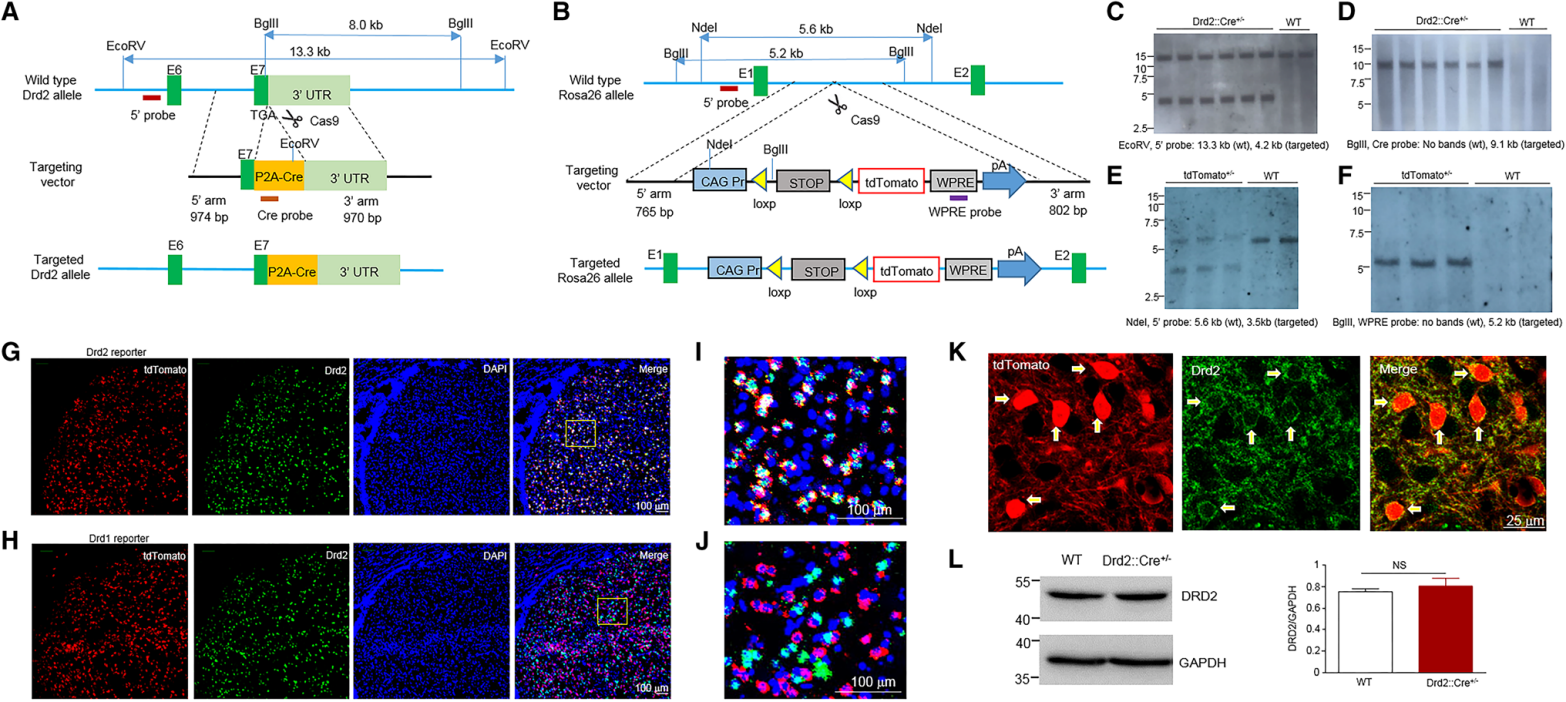
Generation of Drd2::Cre and Rosa26-LSL-tdTomato knockin rats and verification of Drd2 reporter rats. **a** Schematic diagram of the gene targeting strategy to insert the p2A-Cre cassette immediately before the stop codon of the Drd2 locus, between exons 7 and 3′ untranslational region (3′UTR). The p2A peptide will be cleaved and two independent protein DRD2 and CRE will be expressed. **b** Schematic diagram of the gene targeting strategy to insert the Cre reporter cassette into the Rosa26 locus between exon 1 and 2. The Cre reporter cassette is composed of CMV-IE enhancer/chicken β-actin/rabbit β-globin hybrid (CAG) promoter, a loxP (yellow triangles) flanked stop cassette, tdTomato red florescent protein, a woodchuck hepatitis virus post-translational regulatory element (WPRE), and a poly A tail. **c** Southern blot screen using EcoRV-digested genomic DNA and the 5′ probe indicated in the **a**. The wild-type and targeted Drd2 allele will yield a DNA fragment of 13.3 kb and 4.2 kb, respectively. **d** Southern blot screen using BglII-digested genomic DNA and the Cre probe indicated in the **a**. The targeted Drd2 allele will yield a DNA fragment of 9.1 kb. **e** Southern blot screen using Nde1-digested genomic DNA and the 5′ probe indicated in the **d**. The wild-type and targeted Rosa 26 allele will yield a DNA fragment of 5.6 kb and 3.5 kb, respectively. **f** Southern blot screen using BglII-digested genomic DNA and the WPRE probe indicated in the **d**. The targeted Rosa 26 allele will yield a DNA fragment of 5.2 kb. **g** Double fluorescence in situ hybridization (dFISH) of tdTomato and Drd2 mRNA in the striatum of Drd2 reporter rats. Scale bar, 100 µ m. **h** dFISH of tdTomato and Drd2 mRNA in the striatum of Drd1 reporter rats. Scale bar, 100 µm. **i** The enlarged image from the rectangle in **g**. Scale bar, 100 µm. **j** The enlarged image from the rectangle in **h**. Scale bar, 100 µm. **k** Immunofluorescent images of tdTomato and DRD2 in the striatum of Drd2 reporter rats. Arrows indicate the colocalization of tdTomato and DRD2. Scale bar, 25 µm. **l** Western blot of DRD2 and GAPDH in the striatum from Drd2::Cre^+/−^ and WT rats. Left, representative blots, right, quantification results. *NS* not significant, *n* = 3, *t* test

We generated Drd2 reporter rats which expressed tdTomato specifically in Drd2^+^ cells by crossing Drd2::Cre knockin rats (Fig. 1a), where the expression of Cre recombinase is under the control of endogenous Drd2 promoter, with Rosa26::LSL-tdTomato knockin rats (Fig. 1b). The Drd2 reporter rats develop normally and are fertile. Southern blot results validated the insertion into the target locus, indicating that the rat lines are methodologically reliable (Fig. 1c–f). To verify that tdTomato is specifically and faithfully expressed in Drd2 but not Drd1-positive cells, we used fluorescence in situ hybridization (FISH) to detect the mRNA of Drd2 in the striatum where the expression of Drd2 and Drd1 is abundant and separated (Gerfen et al. 1990). As shown in Fig. 1g, i, Drd2 mRNA was well colocalized with tdTomato in the striatum of Drd2 reporter rats. Most tdTomato-positive cells (95 ± 1.5%) from Drd2 reporter striatum express Drd2 mRNA. By contrast, we barely observed colocalization between Drd2 mRNA and tdTomato in the striatum from Drd1 reporter rats (Fig. 1h, j). Only a minority of tdTomato-positive cells (5 ± 1.3%) from Drd1 reporter striatum express Drd2 mRNA. To corroborate the results form in situ hybridization, we used antibody labeling to show the expression of DRD2 protein in the tdTomato-positive cells (Fig. 1k). In addition, the protein levels of DRD2 are similar between the Drd2::Cre^+/−^ rats and their control littermates (Fig. 1l). Thus, we validated on mRNA and protein levels that tdTomato from Drd2 reporter rats can be used as a faithful indicator of Drd2^+^ neurons.

### The expression pattern of Drd2 in adult rat forebrain

We first studied the expression pattern of Drd2 in the olfactory bulb, cerebral cortex, and hippocampus from adult (2-month-old) rats.

#### Olfactory bulb

**Fig. 2:**
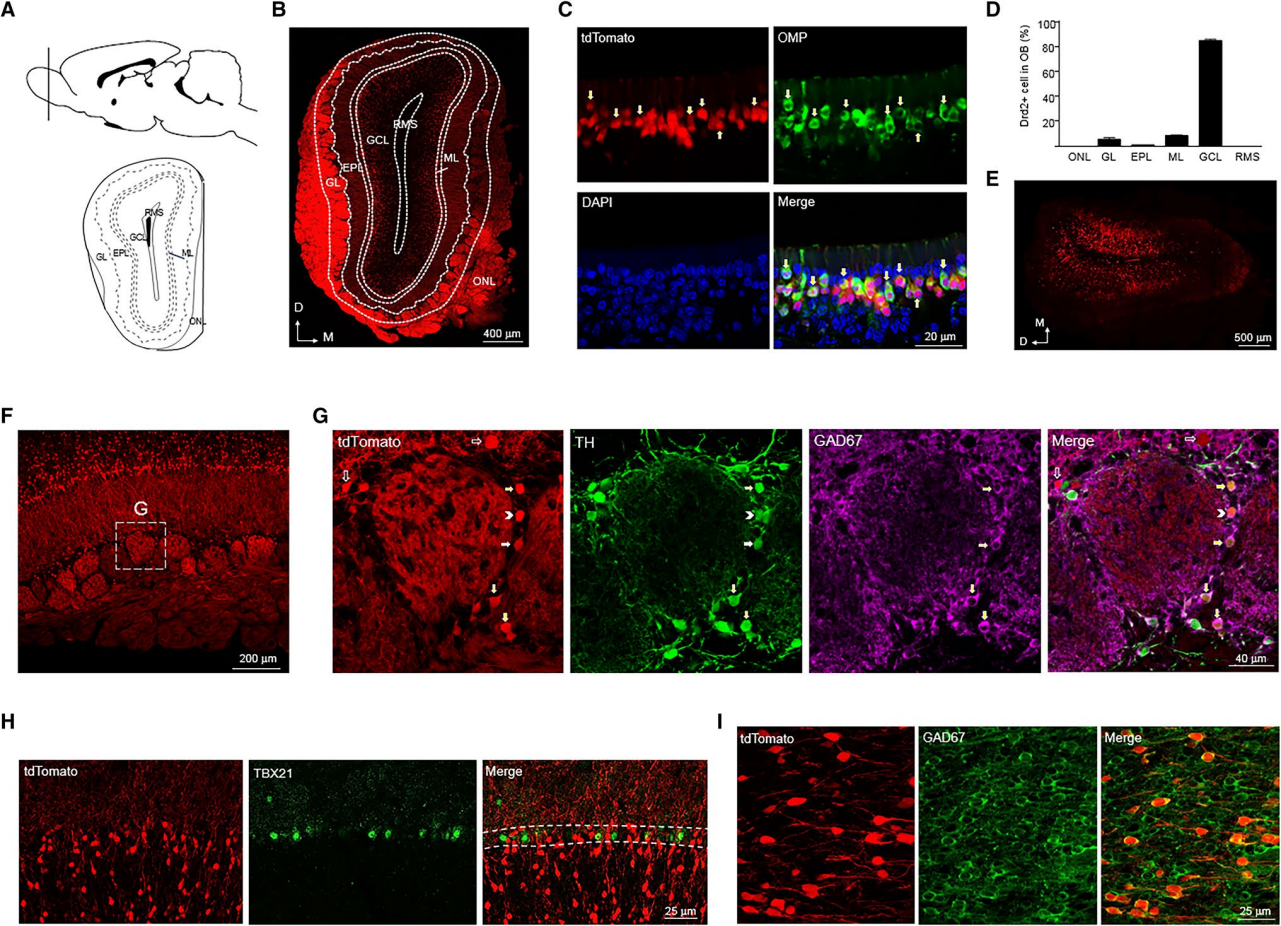
Expression of tdTomato in the olfactory bulb (OB) of adult Drd2 reporter rats. **a** Diagram of rat brain sagittal section (top) and coronal section (bottom). The dashed line of the sagittal section diagram indicates the position of the coronal section. **b** Expression of tdTomato in OB. Scale bar, 400 µm. **c** Immunofluorescent image of tdTomato, OMP, and DAPI in the olfactory epithelial layer. Arrows indicate Drd2^+^ olfactory sensory neurons. Scale bar, 20 µm. **d** The percentage of Drd2^+^ cells in different layers among total Drd2^+^ cells in the OB. **e** Expression of tdTomato when injection of AAV-LSL-tdTomato into the GCL of adult Drd2::Cre rats. Scale bar, 500 µm. **f** Expression of tdTomato in GL. Scale bar, 200 µm. **g** Immunofluorescent image of tdTomato, TH, and GAD67 from the rectangle in **f**. Scale bar, 40 µm. Arrows indicate Drd2^+^ cells expressing both TH and GAD67, arrowheads indicate Drd2^+^ cells only expressing TH and empty arrows indicate Drd2^+^ cells expressing neither TH nor GAD67. **h** Immunofluorescent image of tdTomato and TBX 21 in the MCL. Scale bar, 25 µm. **i** Immunofluorescent image of tdTomato and GAD67 in the GCL. Scale bar, 25 µm. *ONL* olfactory nerve layer, *GL* glomerular layer, *EPL* external plexiform layer, *MCL* mitral cell layer, *GCL* granular cell layer, *RMS* rostral migratory stream

The olfactory bulb (OB) is the first center processing olfactory information and hosts the most numerous dopaminergic neurons in the mammalian central nervous system (Cave and Baker 2009). The OB can be divided into several characteristic layers: olfactory nerve layer (ONL), glomerular layer (GL), external plexiform layer (EPL), mitral layer (ML) and granule cell layer (GCL) (Fig. 2a).

We found strong tdTomato expression in the GL (Fig. 2b), where olfactory sensory neurons (OSN) synapse with mitral cells from the ML. We also observed strong tdTomato expression in the ONL (Fig. 2b), suggesting that the GL labeling comes from coalesced OSN axons. Likewise, Drd2 is strongly expressed in most of the mature OSN in the olfactory epithelium (Fig. 2c). The Drd2^+^ cells were most abundant in the GCL and were also distributed in the GL and ML, while Drd2^+^ cells in the EPL and rostral migratory stream (RMS) were sparse (Fig. 2b, d).

The adult expression of Drd2 in the granule cells of OB is not reported by the previous literature, which raised a possibility that the tdTomato signal which we observed may arise from the transient expression of Drd2 during the early development but not in adulthood. To address this issue, we did stereotaxic injection of AAV carrying the loxp–stop–loxp (LSL)–tdTomato cassette into the GCL of 2-month-old control and Drd2::Cre rats. TdTomato was not expressed in the granule cells when the AAV viruses were injected into the control rats (data not shown). However, we observed the expression of tdTomato in the granule cells when the AAV were injected into the Drd2::Cre rats (Fig. 2e). These results suggest that Drd2 is, indeed, expressed in the OB granule cells of adult rats.

Drd2 was also expressed in juxtaglomerular cells (JGCs) surrounding glomerulus (Fig. 2f, g). Among the Drd2^+^ ventrolateral JGCs, some were short axon cells (SACs) expressing both TH and GAD67; others were GABAergic interneurons expressing GAD67 and a few of them express neither TH nor GAD67 (Fig. 2g). Drd2 was not expressed in mitral cells as we did not observe the colocalization of tdTomato and TBX21, a mitral cell marker (Fig. 2h). In the GCL, Drd2 was mainly expressed in the GABAergic interneurons (Fig. 2i), consistent with the fact that most of the neurons in the GCL are GABAergic interneurons.

#### Cerebral cortex

In addition to regulating the activity of striatum, dopamine signaling also modulates cortical function (Puig et al. 2014). The cerebral cortex is the outer most structure of the mammalian brain having a distinct six-layer composition. Here, high-level processing occurs for many processes including motor control, sensory perception, attention, and memory. The Drd2^+^ cells were widely distributed in different cortical regions.

**Fig. 3:**
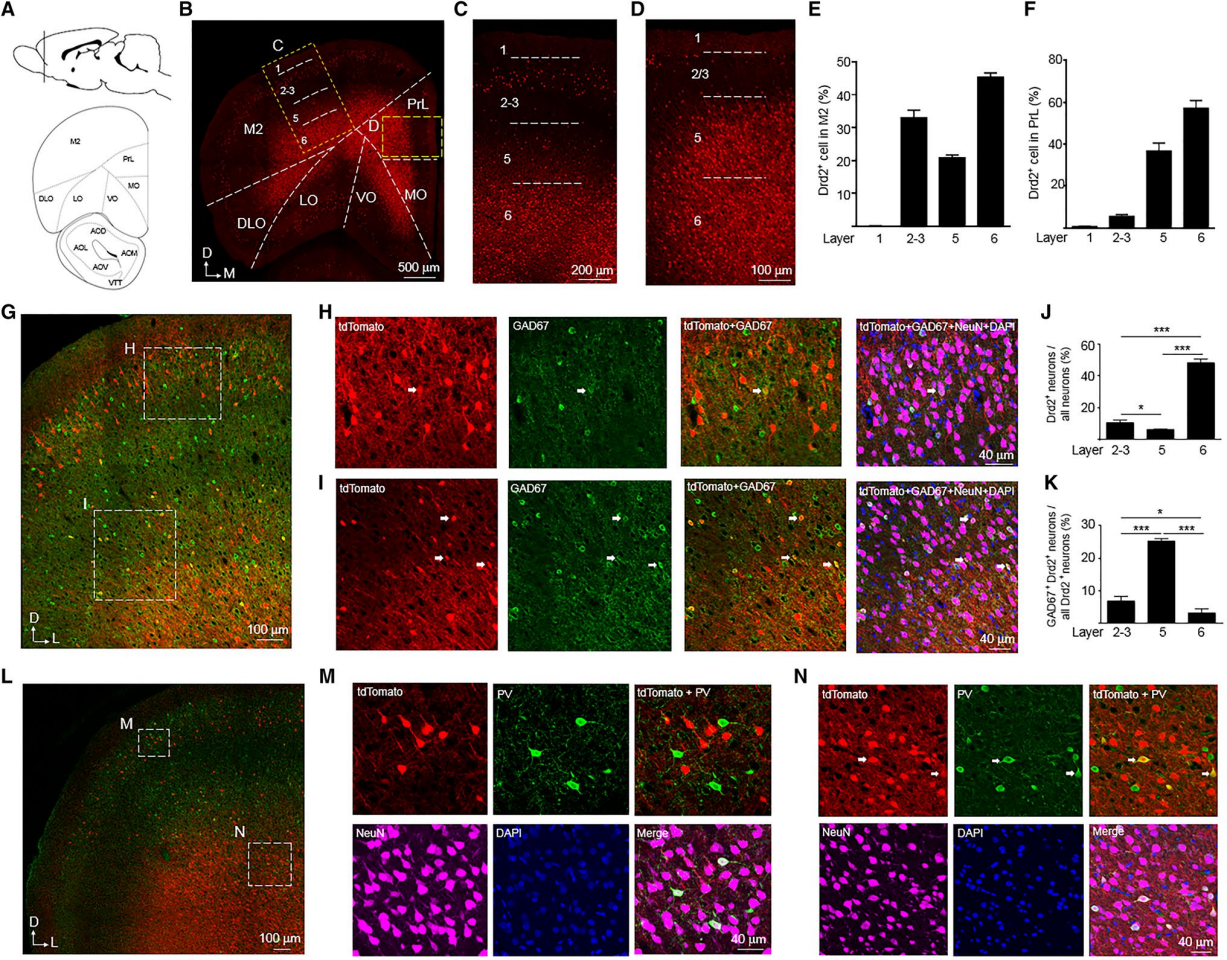
Expression of tdTomato in the prefrontal cortex (PFC) of Drd2 reporter rats. **a** Diagram of rat brain sagittal section (top) and coronal section (bottom). The dashed line of the sagittal section diagram indicates the position of the coronal section. **b** Expression of tdTomato in the PFC. Scale bar, 500 µm. **c, d** Enlarged images from the rectangles in **b**. Scale bar, 200 µm for **c**, 100 µm for **d. e** The percentage of Drd2^+^ cells in different layers among total Drd2^+^ cells in the M2. **f** The percentage of Drd2^+^ cells in different layers among total Drd2^+^ cells in the PrL. **g** Expression of tdTomato and GAD67 in M2. Scale bar, 100 µm. **h, i** Immunofluorescent images of tdTomato, GAD67, NeuN, and DAPI from the rectangles in panel G. Arrows indicate Drd2^+^ GABAergic interneurons. Scale bar, 40 µm. **j** The percentage of Drd2^+^ neurons among all neurons in the M2. ***p < 0.001, *n* = 3, *t* test. **k** The percentage of GABAergic interneurons in the Drd2^+^ neurons in the M2. ***p < 0.001, *n* = 3, *t* test. **l** Expression of tdTomato and PV in M2. Scale bar, 100 µm. **m, n**, Immunofluorescent images of tdTomato, PV, NeuN, and DAPI from the rectangles in panel L. Arrows indicate Drd2^+^ PV-positive interneurons. Scale bar, 40 µm. *M2* secondary motor cortex, *PrL* prelimbic cortex, *MO* medial orbital cortex, *VO* ventral orbital cortex, *LO* lateral orbital cortex, *DLO* dorsolateral orbital cortex

The prefrontal cortex (PFC) is involved in various higher order brain functions, many of which are altered in psychiatric diseases (Lewis and Sweet 2009). We did not observe any Drd2^+^ cells in the layer 1 of rat PFC (Fig. 3a–f). More Drd2^+^ cells were distributed in the layer 6 than layer 2–3 and 5 in the M2 of rat PFC (Fig. 3a–e). Consistent with this result was the observation that higher percentage of neurons express Drd2 in the layer 6 compared with layer 2–3 and 5 (Fig. 3g–j). Similar with the M2, the PrL has the highest number of Drd2^+^ cells in the layer 6 (Fig. 3b, d, f). However, more Drd2^+^ cells were found in the layer 5 than 2–3 in the PrL, which is opposite to the M2 (Fig. 3b–f). Higher percentage of Drd2^+^ neurons were GABAergic interneurons in the layer 5 than layer 2–3 and 6 of M2 (Fig. 3g–i, k). The GABAergic interneurons expressing Drd2 in the layer 2–3 of M2 were parvalbumin (PV)-negative (Fig. 3l, m). By contrast, the Drd2^+^ GABAergic interneurons appeared to express PV in the layer 5 of M2 (Fig. 3l, n).

**Fig. 4:**
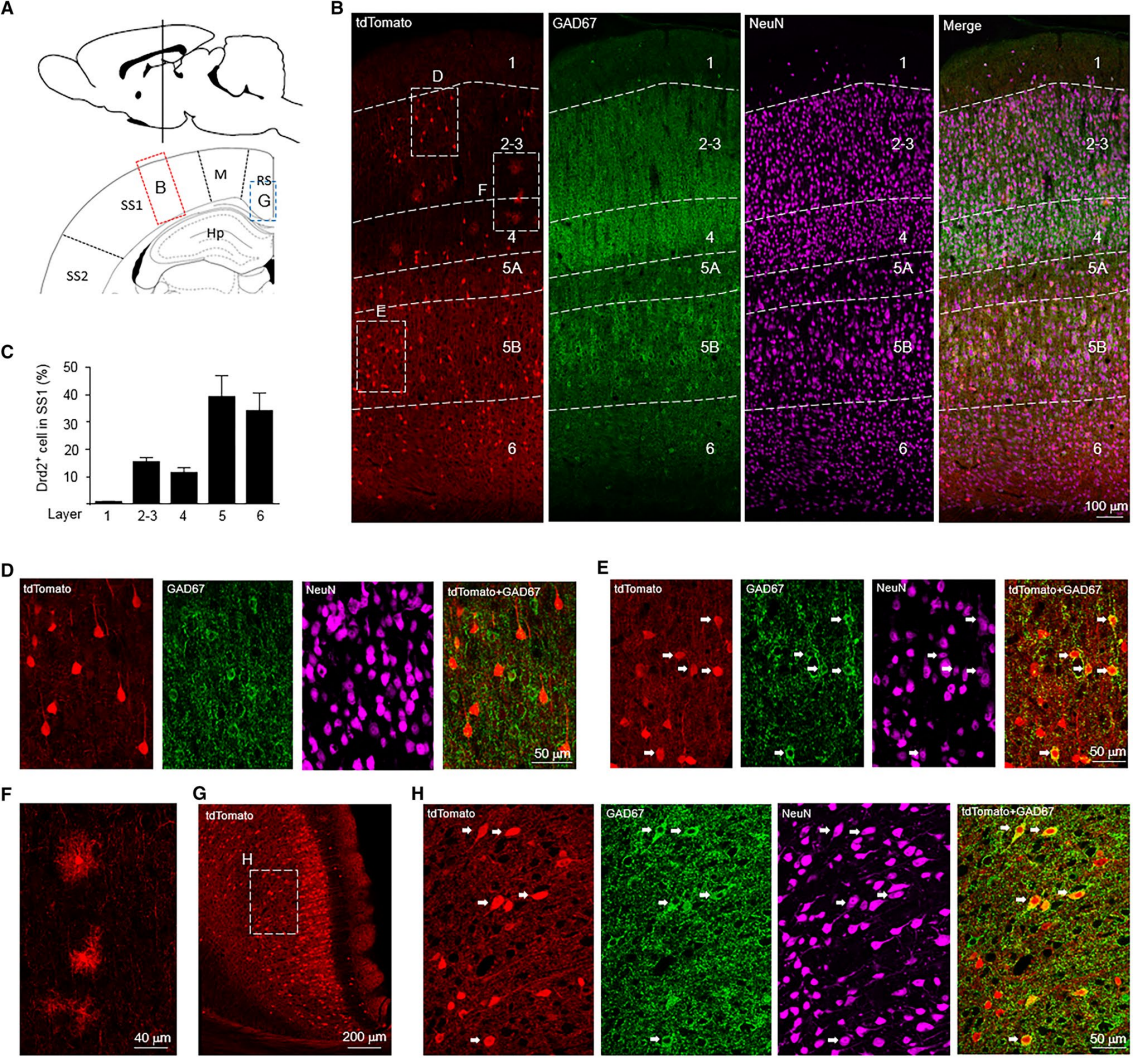
Expression of tdTomato in the SS1 and RS of Drd2 reporter rats. **a** Diagram of rat brain sagittal section (top) and coronal section (bottom). The dashed line of the sagittal section diagram indicates the position of the coronal section. The red and blue rectangles indicate the brain regions shown in **b, g. b** Immunofluorescent images of tdTomato, GAD67 and NeuN in SS1. Scale bar, 100 µm. **c** The percentage of Drd2^+^ cells in different layers among total Drd2^+^ cells in the SS1. **d, e** Immunofluorescent images of tdTomato, GAD67, and NeuN from the rectangles in **b**. Arrows indicate Drd2^+^ GABAergic interneurons. Scale bar, 50 µm. **f** Enlarged image from the rectangle in **b**. Scale bar, 40 µm. **g** Expression of tdTomato in RS. Scale bar, 200 µm. **h** Immunofluorescent images of tdTomato, GAD67, and NeuN from the rectangle in **g**. Arrows indicate Drd2^+^ GABAergic interneurons. Scale bar, 50 µm. SS1: primary somatosensory cortex, *RS* retrosplenial cortex

In addition, we found a moderate number of Drd2^+^ cells in the primary somatosensory cortex (SS1). Drd2^+^ cells were evenly distributed in the layer 2–4, 5, and 6 (Fig. 4a–c). Most Drd2^+^ cells (95.9 ± 1.0%) in the layer 2 of SS1 were putative pyramidal neurons as indicated by the colocalization of tdTomato with NeuN but not GAD67 (Fig. 4b, d). Strikingly, Drd2 appeared to be expressed in glia cells in the layer 3 and 4 of SS1 (Fig. 4b, f). By contrast, about half Drd2^+^ cells (50.1 ± 2.8%) were GABAergic interneurons in the layer 5 of SS1 (Fig. 4b, e). Likewise, in the deep layer of retrosplenial cortex (RS), 25.5 ± 3.2 percentage of Drd2^+^ cells are GABAergic interneurons (Fig. 4g, h).

**Fig. 5:**
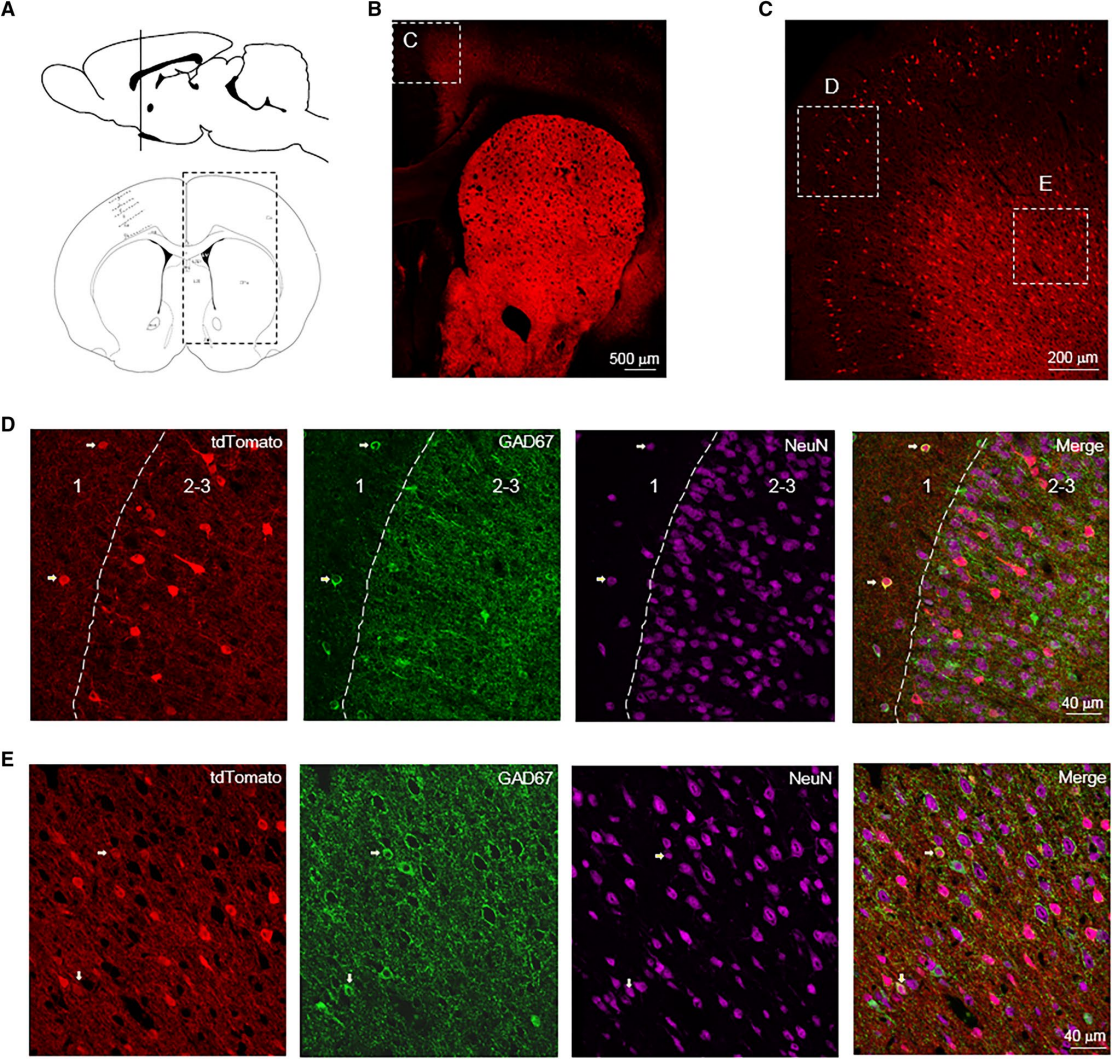
Expression of tdTomato in the ACC of Drd2 reporter rats. **a** Diagram of rat brain sagittal section (top) and coronal section (bottom). The dashed line of the sagittal section diagram indicates the position of the coronal section. The rectangle indicates the brain regions shown in **b. b** Expression of tdTomato. Scale bar, 500 µm. **c** Expression of tdTomato from the rectangle in **b**. Scale bar, 200 µm. **d, e** Immunofluorescent images of tdTomato, GAD67, and NeuN from the rectangles in **c**. Arrows indicate Drd2^+^ GABAergic interneurons. Scale bar, 40 µm. *ACC* anterior cingulate cortex

The anterior cingulate cortex (ACC) is connected to the prefrontal cortex and is involved in high-level functions such as cognition and emotion (Apps et al. 2016; Shackman et al. 2011). Drd2 was only expressed in GABAergic interneurons in the layer 1 of ACC (Fig. 5a–d). However, most Drd2^+^ cells (91.3 ± 2.3%) were putative pyramidal neurons in the layer 2–3 of ACC as indicated by the colocalization of tdTomato with NeuN but not GAD67 (Fig. 5a–d). Likewise, only a minority of Drd2^+^ cells (7.5 ± 0.3%) were GABAergic interneurons in the layer 5–6 of ACC (Fig. 5e).

#### Hippocampus

**Fig. 6:**
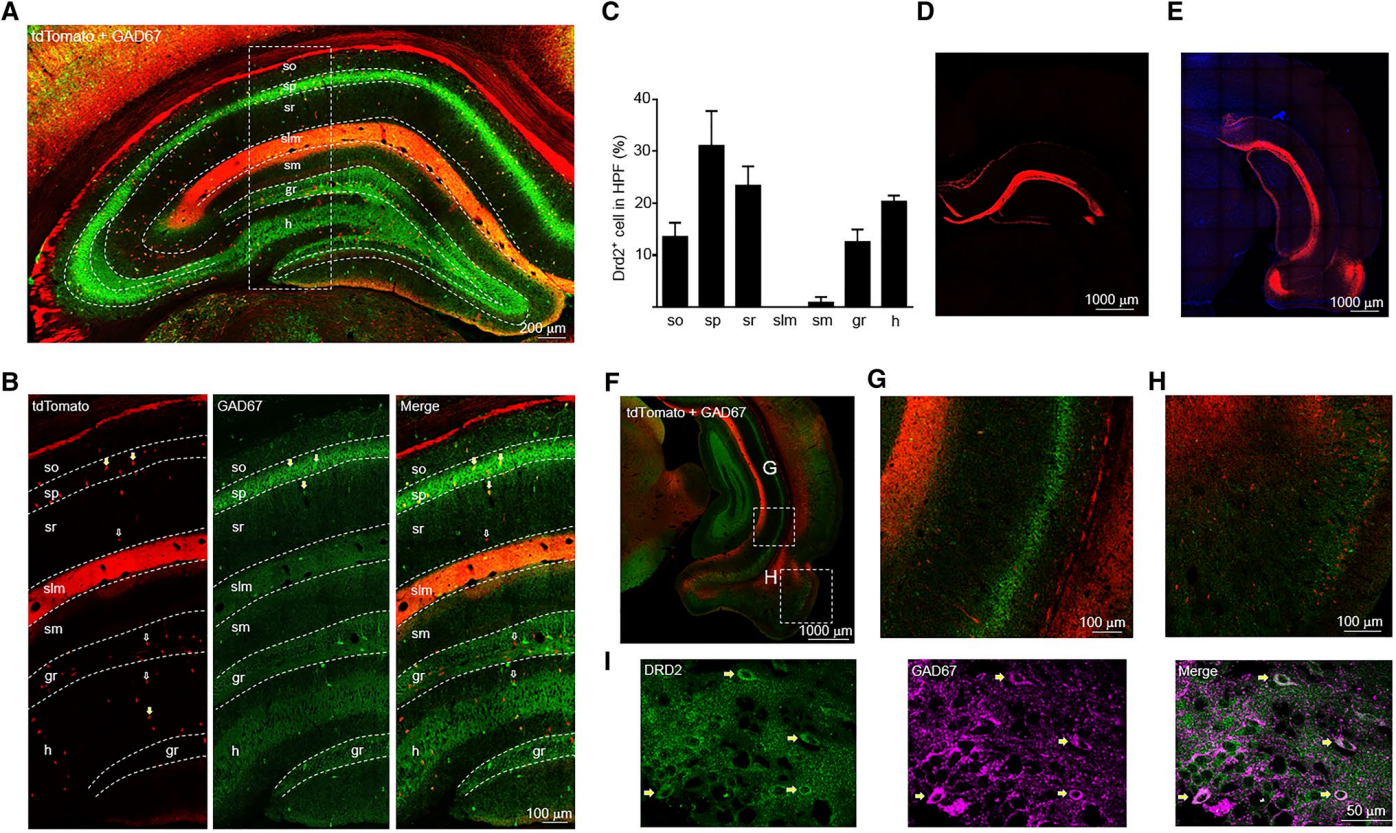
Expression of tdTomato in the hippocampus of Drd2 reporter rats. **a** Immunofluorescent images of tdTomato and GAD67 in the dorsal hippocampus. Scale bar, 200 µm. **b** Enlarged images of the rectangle in **a**. Arrows indicate Drd2^+^ GABAergic interneurons. Empty arrows indicate Drd2^+^ cells negative for GAD67. Scale bar, 100 µm. **c** The percentage of Drd2^+^ cells in different layers among total Drd2^+^ cells in the dorsal hippocampus. Expression of tdTomato in the slm of CA1 region of dorsal (**d**) and ventral (**e**) hippocampus after injecting AAV-LSL-tdTomato into the entorhinal cortex. Scale bar, 1000 µm. **f** Expression of tdTomato in the ventral hippocampus. Scale bar, 1000 µm. **g, h** Enlarged images of the rectangles in **f**. Scale bar, 100 µm. **i** Immunofluorescent images of DRD2 and GAD67 in the ventral hippocampus. Arrows indicate Drd2^+^ GABAergic interneurons. Scale bar, 50 µm. *so* stratum oriens, *sp* stratum pyramidale, *sr* stratum radiatum, *slm* stratum lacunosum, *osm* outer stratum moleculare, *ism* inter stratum moleculare, *gr* granule cell layer, *h* hilus

The hippocampus, located beneath the cerebral cortex, is critically involved in learning and memory, in addition to spatial navigation (Squire 1992). The hippocampus can be divided into cornu ammonis (CA) 1, 2, and 3 areas and the dentate gyrus (DG). Our results indicated that Drd2 was mainly expressed in the stratum pyramidale (sp) and stratum radiatum (sr) of CA1-3 regions and the DG (Fig. 6a, c). In the sp of CA1-3 regions, most Drd2^+^ cells were GABAergic interneurons as shown by the overlay between tdTomato and GAD67 (Fig. 6a, b). Strikingly, we observed strong tdTomato expression in the stratum lacunosum moleculare (slm) of CA1 region (Fig. 6a). However, tdTomato seemed to be expressed in axon terminals innervating the slm rather than in cell bodies (Fig. 6b). The slm of CA1 region receive glutamatergic input from layer 2 island cells in the entorhinal cortex (EC) (Kitamura et al. 2014). We speculate that Drd2^+^ neurons in the EC send their axon projections to the slm of CA1 region. To examine this possibility, we did stereotaxic injections of AAV encoding LSL-tdTomato into the EC of adult Drd2::Cre rats. This revealed strong tdTomato expression in the slm of CA1 region (Fig. 6d, e). These results indicate that tdTomato in the slm of CA1 region originates from the Drd2^+^ pyramidal neurons in the EC. Indeed, Drd2 is expressed in pyramidal neurons of the EC but not of hippocampal CA1 region (Fig. 6e–h).

### Dynamics of Drd2^+^ neurons in the developing forebrain regions

All the above data addressed the expression pattern of Drd2 in the adult rat forebrain. Next, we determined the dynamics of Drd2^+^ neurons in the developing forebrain regions including OB, cerebral cortex, and hippocampus. The volume of forebrain regions increases and the neuronal number is dynamically changed during postnatal brain growth in rats (Bandeira et al. 2009). Due to these reasons, the percentage of Drd2^+^ neurons in total neurons may better reflect the profiles of Drd2 expression during postnatal development. To this end, we compared the percentage of Drd2^+^ neurons in OB and cerebral cortex from different aged Drd2 reporter rats.

#### Olfactory bulb

**Fig. 7:**
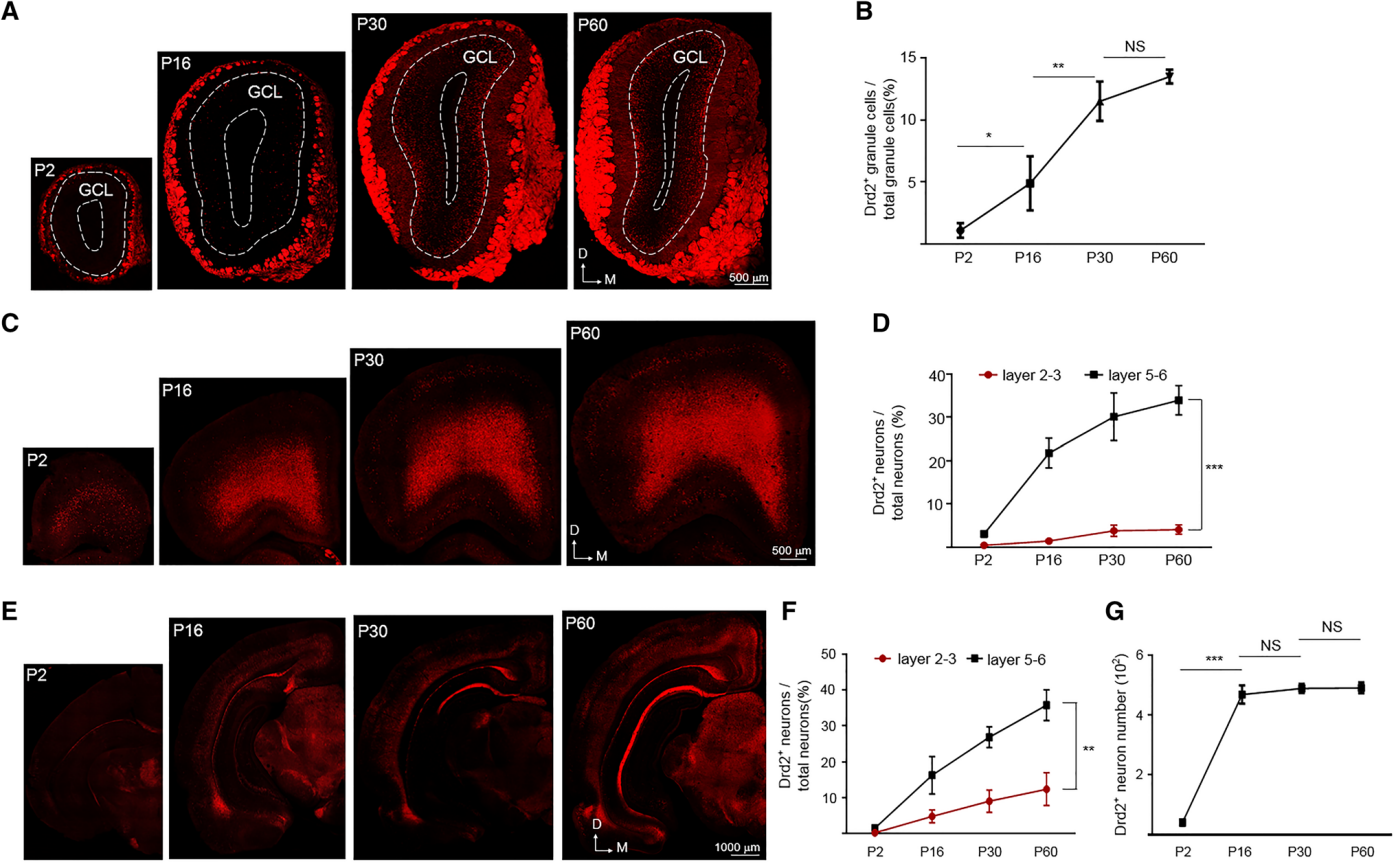
The Drd2^+^ cells in the olfactory bulb (OB), prefrontal cortex (PFC), second visual cortex (V2), entorhinal cortex (EC), and hippocampus of different aged Drd2 reporter rats. **a** Expression of tdTomato in the OB. Scale bar, 500 µm. **b** The percentage of Drd2^+^ granule cells among total granule cells in the OB. *NS* not significant, **p < 0.01, *p < 0.05, *n* = 3, one-way ANOVA. **c** Expression of tdTomato in the PFC. Scale bar, 500 µm. **d** The percentage of Drd2^+^ neurons among total neurons in the layer 2–3 and 5–6 of PFC. ***p < 0.001, *n* = 3, two-way ANOVA. **e** Expression of tdTomato in the V2, EC and hippocampus. Scale bar, 1000 µm. **f** The percentage of Drd2^+^ neurons among total neurons in the layer 2–3 and 5–6 of V2. **p < 0.01, *n* = 3, two-way ANOVA. **g** the number of Drd2^+^ neurons in the ventral hippocampus as indicated in **e**. *NS* not significant, ***p < 0.001, *n* = 3, two-way ANOVA

In the P2 olfactory bulb, Drd2 was only expressed in the GL but not the GCL (Fig. 7a). Since tdTomato in the GL is from the projections of OSN, this result suggested that the expression of Drd2 in OSN was earlier than that in granule cells. The expression of Drd2 in the GCL appeared at P16 and reached the adult levels at P30 (Fig. 7a, b). The percentage of Drd2^+^ neurons in total neurons (as indicated by NeuN staining, data not shown) in the GCL is similar between P60 and P30 (Fig. 7a, b).

#### Cerebral cortex

The percentage of Drd2^+^ neurons in the layer 2–3 of PFC is lower than that in the layer 5–6 (Fig. 7c, d) consistent with the previous data (Fig. 3a–f). However, the percentage of Drd2^+^ neurons among total neurons in both superficial and deep layer of PFC increased from P2 to P30, and then remains stable from P30 to adult (Fig. 7c, d). In the second visual cortex (V2), the percentage of Drd2^+^ neurons in the layer 2–3 is also lower than that in the layer 5–6 (Fig. 7e, f). However, the percentage of Drd2^+^ neurons in both superficial and deep layers of V2 increased gradually from P2 to adulthood (Fig. 7e, f). Neurons in the layer 2–3 of EC appear to express Drd2 after P16 and neurons express Drd2 in the EC at P30 and P60 (Fig. 7e). In agreement, the projections of Drd2^+^ neurons from the EC to the slm of CA1 region (indicated by tdTomato in the slm) were observed at P30 and P60 but not at P2 or P16 (Fig. 7e).

#### Hippocampus

Since Drd2 is only expressed in GABAergic interneurons in the hippocampus, the percentage of Drd2^+^ neurons is too low to be counted in the hippocampus. Due to these reasons, we quantified the number of Drd2^+^ neurons in the ventral hippocampus during postnatal development. As shown in Fig. 7e, g, the Drd2^+^ neuron number in the ventral hippocampus increased significantly from P2 to P16 and kept constant during P16 and P60.

### Different expression pattern of tdTomato between Drd2 reporter rats and mice

**Fig. 8:**
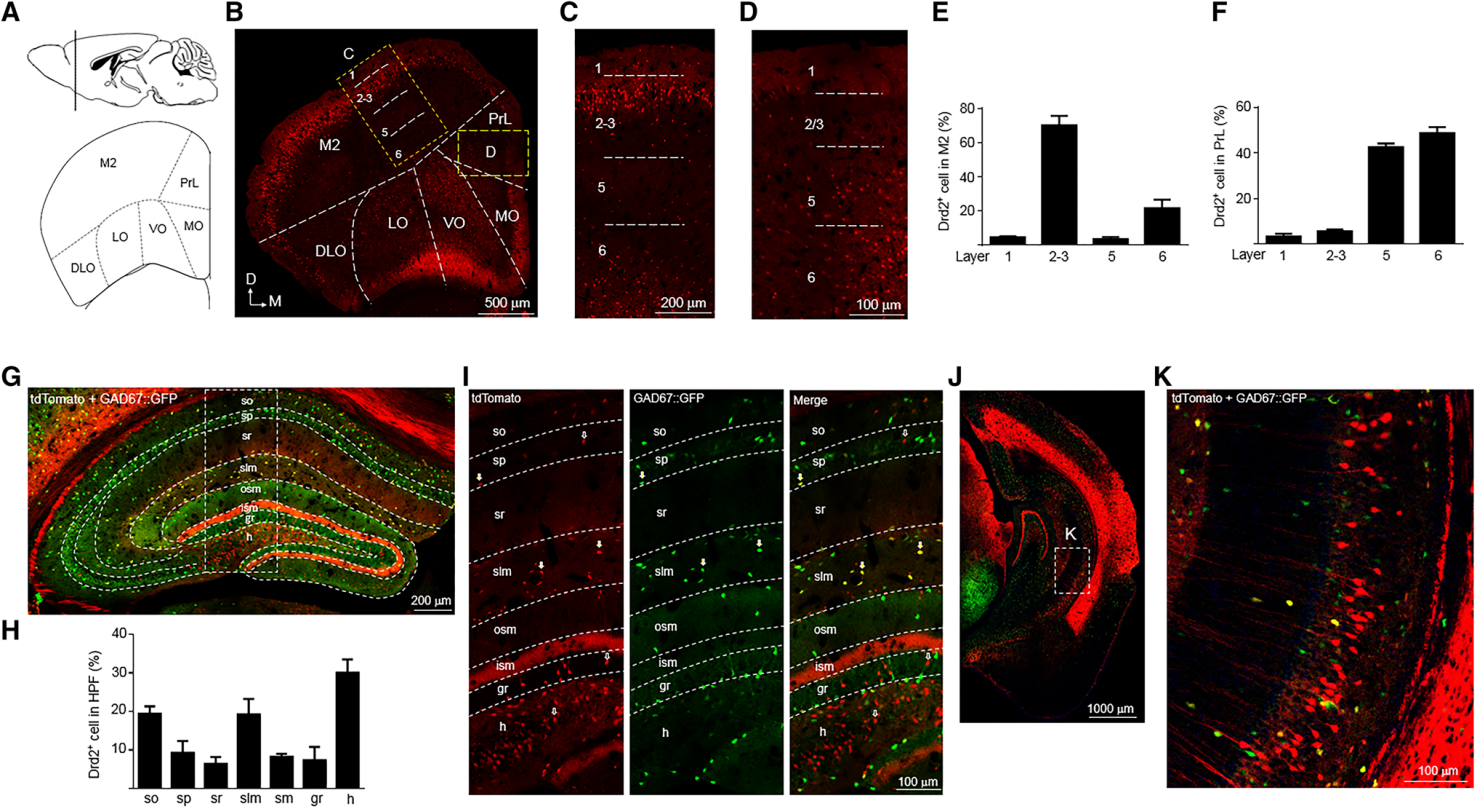
Expression of tdTomato in the PFC and hippocampus of adult Drd2 reporter mice. **a** Diagram of mouse brain sagittal section (top) and coronal section (bottom). The dashed line of the sagittal section diagram indicates the position of the coronal section. **b** Expression of tdTomato in the PFC of Drd2 reporter mice. Scale bar, 500 µm. **c, d** Enlarged images from the rectangles in **b**. Scale bar, 200 µm for **c**, 100 µm for **d. e** The percentage of Drd2^+^ cells in different layers among total Drd2^+^ cells in the M2. **f** The percentage of Drd2^+^ cells in different layers among total Drd2^+^ cells in the PrL. **g** Immunofluorescent images of tdTomato and GAD67::GFP in the dorsal hippocampus. The Drd2 reporter mice were crossed with GAD67::GFP mice to obtain this figure. Scale bar, 200 µm. **h** The percentage of Drd2^+^ cells in different layers among total Drd2^+^ cells in the dorsal hippocampus. **i** Enlarged images of the rectangle in **g**. Arrows indicate Drd2^+^ GABAergic interneurons. Empty arrows indicate Drd2^+^ cells negative for GAD67. Scale bar, 100 µm. **j** Expression of tdTomato and GAD67::GFP in the ventral hippocampus. Scale bar, 1000 µm. **k** Enlarged image from the rectangle in **j**. Scale bar, 100 µm

The Drd2 reporter mice have recently been used to analyze the expression pattern of Drd2 in mouse cortex and hippocampus (Gangarossa et al. 2012; Puighermanal et al. 2015; Wei et al. 2018). In the following study, we sought to determine whether the expression pattern of Drd2 is similar between Drd2 reporter rats and mice in the PFC and hippocampus. In the M2 from Drd2 reporter mice, more Drd2^+^ cells were distributed in layer 2–3 than layer 5–6 (Fig. 8a–c, e), which is opposite to Drd2 reporter rats. Drd2^+^ cells were found in the layer 1 of M2 from Drd2 reporter mice (Fig. 8c) but not Drd2 reporter rats (Fig. 3c). In the prelimbic cortex (PrL), the percentage of Drd2^+^ cells in different layers was similar between Drd2 reporter rats (Fig. 3a, b, d, f) and mice (Fig. 8a, b, d, f).

Drd2 was mainly expressed in the hilar mossy cells in Drd2 reporter mice as shown by tdTomato in the soma and their projections to the ism of DG (Fig. 8g). In addition to the hilus of DG, Drd2^+^ cells were also found in the other layers of dorsal hippocampus (Fig. 8g, h). Consistent with the previous findings, the majority of Drd2^+^ cells in the CA1-3 regions of dorsal hippocampus were GABAergic interneurons in Drd2 reporter mice (Fig. 8g, i). In Drd2 reporter mice, Drd2 was not expressed in pyramidal neurons of the EC (Fig. 8j), and thus, unlike what we found in Drd2 reporter rats, we did not observe tdTomato in the slm of CA1 region (Fig. 8g, i, j). By contrast, we observed Drd2 expression in pyramidal neurons of the CA1 region in the ventral hippocampus from Drd2 reporter mice (Fig. 8j, k) but not Drd2 reporter rats (Fig. 6f, g, i).

## Discussion

The present study demonstrated the cellular expression pattern of D2 dopamine receptor in adult and postnatal rat forebrain. Here, we discuss the new information revealed by the Drd2 reporter rats and the relevance to physiology and schizophrenia.

Drd2 is highly expressed in the granule cells of the olfactory bulb. The reciprocal dendro-dendritic synapses formed between mitral and granule cell are considered to be important for the synchronization of mitral cells, affecting the ability of odor discrimination (Schoppa 2006; Shepherd et al. 2007). Intriguingly, olfactory discrimination deficits were observed in Drd2 null mutant mice (Tillerson et al. 2006). However, blockade of Drd2 but not Drd1 in the olfactory bulb improved odor discrimination in adult rats (Escanilla et al. 2009). These studies suggest the importance of Drd2 in odor discrimination, although the underlying mechanisms are not clear. Since our results indicated the expression of Drd2 in granule cells but not mitral cells, it might be possible that Drd2 in granule cells is important for odor discrimination. In support of this hypothesis is the dramatic increase of Drd2 expression in granule cells from the early postnatal stage to P30, which is consistent with the time course of maturation of odor discrimination in rats (Gregory and Pfaff 1971; Salas et al. 1970).

Drd2 is enriched in EC pyramidal neurons which project to the slm of CA1 region. A recent study suggests that island cells in the layer 2 of EC directly project to the slm of CA1 region and control trace fear conditioning (Kitamura et al. 2014). Thus, we speculate that Drd2 might be important for normal function of island cells and as such might have a role in trace fear conditioning. Consistent with this hypothesis is the previous study, demonstrating that activation of Drd2 reduced fear expression in rats (de Oliveira et al. 2006). Intriguingly, our results indicated that EC Drd2^+^ neurons and their projection to CA1 region rapidly increased during postnatal period, which concur with the age-related increase in rats’ abilities of fear learning as the trace interval was lengthened (Moye and Rudy 1987).

In the PFC, the percentage of Drd2 + neurons dramatically increase from P2 to P30 and remains constant between P30 and P60. Adolescence (around P30 in rodents) is the peak stage of synapse pruning which is abnormally accelerated in schizophrenia (Penzes et al. 2011). A previous study showed that inhibition of Drd2 during the early development increased spine number in the hippocampus (Jia et al. 2013). However, additional experiments are required to demonstrate that reduced Drd2 expression could result in changes in synapse number during development. We revealed that Drd2 was expressed in PV-positive interneurons in the deep but not superficial layer of rat PFC. The dysfunction of PV-positive interneurons in PFC is considered an important pathophysiological mechanism of schizophrenia (Lewis and Sweet 2009; Wen et al. 2010; Yin et al. 2013b). Interestingly, the previous studies showed that Drd2 was important for the function of GABAergic interneurons in the deep layer of rat PFC (Tseng and O’Donnell 2007; Xu and Yao 2010). A recent paper indicated that deletion of Drd2 from PV-positive interneurons resulted in schizophrenia-like phenotypes in mice (Tomasella et al. 2018).

Our results may help to understand the cellular mechanisms underlying how Drd2 signaling modulates synaptic plasticity in the hippocampus. Genetic deletion or pharmacological inhibition of Drd2 prevents both LTP and LTD in CA1 pyramidal neurons (Frey and Matthies 1990). In the dentate gyrus, blockade of Drd2 inhibits LTP both in vivo and in hippocampal slices (Manahan-Vaughan and Kulla 2003). Our data and previous studies (Gangarossa et al. 2012; Puighermanal et al. 2015) indicate that Drd2 is mostly expressed in GABAergic interneurons in the hippocampus, which supports the hypothesis that Drd2 regulates hippocampal LTP through GABAergic interneurons. Since Drd2 activation reduces GABA synthesis in the hippocampus (Steulet et al. 1990), blockade of Drd2 might cause enhanced GABA transmission and prevention of LTP induction. Consistent with this hypothesis are the previous studies, demonstrating that enhanced GABA transmission causes reduced LTP (Chen et al. 2010; Wigstrom and Gustafsson 1983).

Of note, the Drd2 expression pattern is distinctive between Drd2 reporter rats and mice in the PFC and hippocampus, two brain regions implicated in the pathophysiology of schizophrenia (Harrison 2004; Lewis and Sweet 2009). This could reflect the inter-species difference of Drd2 expression between rats and mice. However, the previous studies using ISH and electrophysiology demonstrated that Drd2 was mostly expressed in deep layer neurons in the mouse, rat, and monkey PFC (Gee et al. 2012; Lidow et al. 1998; Santana et al. 2009), which demonstrate the consensus of Drd2 expression among different species and concur with the results from our Drd2 reporter rats. Alternatively, the different expression pattern of Drd2 between Drd2 reporter rats and mice may arise from the distinct strategies in generating these two animal lines. The Drd2::Cre rats were generated by knockin, while the Drd2::Cre mice were constructed by BAC transgene, where the Cre expression pattern might not be the same as the endogenous Drd2 gene. Regardless, the Drd2::Cre rats generated here may become a useful tool to study the function of neuronal populations expressing Drd2.
